# Responsiveness, Minimal Clinically Important Difference, and Validity of the MoCA in Stroke Rehabilitation

**DOI:** 10.1155/2019/2517658

**Published:** 2019-04-14

**Authors:** Ching-Yi Wu, Shuan-Ju Hung, Keh-chung Lin, Kai-Hua Chen, Poyu Chen, Pei-Kwei Tsay

**Affiliations:** ^1^Department of Occupational Therapy and Graduate Institute of Behavioral Sciences, College of Medicine, Chang Gung University, Taoyuan, Taiwan; ^2^Department of Physical Medicine and Rehabilitation, Chang Gung Memorial Hospital at Linkou, Taoyuan, Taiwan; ^3^School of Occupational Therapy, College of Medicine, National Taiwan University, Taipei, Taiwan; ^4^Division of Occupational Therapy, Department of Physical Medicine and Rehabilitation, National Taiwan University Hospital, Taipei, Taiwan; ^5^College of Medicine, Chang Gung University, Taoyuan, Taiwan; ^6^Department of Physical Medicine and Rehabilitation, Chang Gung Memorial Hospital, Chiayi, Taiwan; ^7^School of Nursing, Chang Gung University, Taoyuan, Taiwan

## Abstract

**Objective:**

Persons with stroke frequently suffer from cognitive impairment. The Montreal Cognitive Assessment (MoCA), a recently developed screening tool, is sensitive to poststroke cognitive deficits. The present study assessed its psychometric and clinimetric properties (i.e., responsiveness, minimal clinically important difference (MCID), and criterion validity) in stroke survivors receiving rehabilitative therapy.

**Method:**

The MoCA and the Stroke Impact Scale (SIS) were administered to 65 stroke survivors before and after 4 to 5 weeks of therapy. The effect size and standardized response mean (SRM) were calculated for responsiveness. Anchor- and distribution-based methods were used to estimate the MCID. Criterion validity was measured with the Spearman correlation coefficient.

**Results:**

The responsiveness of the MoCA was moderate (SRM = 0.67). Participants exceeding the MCID according to the anchor- and distribution-based approaches were 33 (50.77%) and 20 (30.77%), respectively. Fair to good concurrent validity was reported between the MoCA and the SIS communication subscale. The MoCA had satisfactory predictive validity with the SIS communication and memory subscales.

**Conclusion:**

This study may support the responsiveness, MCID, and criterion validity of the MoCA in stroke populations. Future studies with larger sample sizes are needed to validate the current findings.

## 1. Introduction

Stroke is one of the leading causes of death and functional disability in adulthood worldwide [1]. Stroke may cause physical impairments [2], and deficits in cognitive domains, including memory, language, attention, and orientation, are also frequently observed [3–5]. Cognitive impairment after stroke is associated with severe functional impairment in daily life activity [5]. To assess the effect of stroke on cognition and evaluate the efficacy of interventions in stroke survivors, the use of instruments with good psychometric or clinimetric characteristics (e.g., reliability, validity, and responsiveness) is essential in research and clinical settings. In this way, stroke survivors' cognitive performance at baseline and change after treatment may be measured and interpreted to inform rehabilitation practice.

The Montreal Cognitive Assessment (MoCA), a brief instrument for screening mild cognitive impairment and dementia [6], assesses several cognition-related domains, including memory, attention, and language, among others (http://www.mocatest.org). The MoCA is widely used to detect cognitive impairment in clinical groups such as patients with Alzheimer disease [7] or with cerebrovascular disease [8]. Earlier studies have pointed out the superiority of the MoCA in screening cognitive impairment after stroke compared with the Mini-Mental State Examination (MMSE) [8, 9]. A population-based study by Pendlebury et al. [8] demonstrated that the MoCA was more able than the MMSE to discriminate between various levels of cognitive ability and to detect more cognitive deficits in patients with transient ischemic attack and stroke. Dong et al. [9] also showed that among people who have experienced transient ischemic attack, the MoCA had higher sensitivity than the MMSE in detecting cognitive deficits, especially in the domains of visuospatial/executive function, attention, and recall. To date, numerous studies focusing on the MMSE and MoCA showed that the MoCA is a valid instrument for detecting cognitive deficits (e.g., [9, 10]).

Poststroke cognitive impairment may influence the quality of life and activities of daily living (ADL) in patients [11]. In the current study, we assessed the criterion validity between the MoCA and domains related to the quality of life by using the Stroke Impact Scale 3.0 (SIS) [12], a stroke-specific measure assessing different aspects of health-related life function, as the criterion measure. As far as we know, this is a first attempt to investigate the validity between the MoCA and the SIS 3.0.

Responsiveness is defined as the ability of an instrument to detect changes over a certain period of time [13]. To our knowledge, only one study [14] has assessed the responsiveness of the MoCA in patients with acquired brain injury (i.e., stroke or traumatic brain injury). Lim et al. [14] reported that the MoCA was moderately responsive to recovery in a subacute sample of 36 patients. The participants in the Lim et al. [14] study was limited to a subacute sample being less than 6 months in stroke onset. The results may not be generalized to chronic patients with stroke onset more than 6 months. Knowledge about the responsiveness of the MoCA in stroke populations is still sparse.

The minimal clinically important difference (MCID) is defined as “the smallest change in score in the domain of interest which patients perceive as beneficial and which would mandate, in the absence of troublesome side effects and excessive cost, a change in the patient's management” [15]. A statistically important change does not correspond to a clinically important change that is meaningful for the interpretation of patient-reported measures [16].

In order to expand knowledge about the psychometric and clinimetric properties of the MoCA in stroke populations, the present study assessed the responsiveness, MCID, and criterion validity of this instrument in patients with stroke receiving rehabilitative therapy.

## 2. Methods

### 2.1. Participants

Stroke survivors were recruited from the departments of rehabilitation at 9 medical centers in Taiwan as part of ongoing studies investigating the effects of rehabilitative treatments on a variety of functions in stroke survivors. The inclusion criteria were (1) age between 20 and 80 years; (2) no severe cognitive deficits, with a score of ≥21 on the MMSE; (3) no severe spasticity in the upper extremity, with a score of <3 on the Modified Ashworth Scale; and (4) ability to follow instructions to complete the assessments and perform therapeutic activity. The institutional review board at each participating center approved the study, and all patients signed the consent forms before they participated in the study.

### 2.2. Procedure

Eligible participants received an intensive 90-minute task-oriented therapy session, 5 times per week, for 4 to 5 weeks. They completed the self-reported SIS 3.0 questionnaire and were evaluated by the occupational therapists using the MoCA before and after the intervention.

### 2.3. Measures

#### 2.3.1. Montreal Cognitive Assessment

The MoCA is a brief standardized instrument for screening cognitive impairment [6]. This study used the Chinese (Taiwan) version of the MoCA version 7 (http://www.mocatest.org). The MoCA measures several domains, including visuospatial, naming, attention, language, abstraction, delayed recall, and orientation. The total scores range from 0 to 30. One point is added to the total scores for participants who received less than 12 years of education. The reliability and validity of the MoCA have been reported in previous studies [17].

#### 2.3.2. Stroke Impact Scale 3.0

The SIS 3.0, a self-reported instrument, has been widely used to assess the quality of life in patients with stroke [12]. It consists of 59 items grouped into 8 subscales: strength, memory, emotion, communication, ADL, mobility, hand function, and social participation. The strength, hand function, and ADL subscales are further grouped into the subscale of physical function. The items are scored on a 5-point scale to indicate the patient's perceived difficulty (5, not difficult at all; 4, a little difficult; 3, somewhat difficult; 2, very difficult; and 1, extremely difficult); thus, higher total scores represent better functioning. The test-retest reliability, validity, and responsiveness of the SIS 3.0 are well established [18].

The SIS 3.0 also contains one question to measure the patient's perceived recovery from stroke, with 0 indicating no recovery and 100 indicating full recovery. The perceived recovery from stroke was chosen as the anchor in the current study when we calculated the MCID estimates because this score directly reflects the perspective of the patients, which is indicated by the studies using the global rating scale as the anchors (e.g., [19]).

### 2.4. Data Analysis

#### 2.4.1. Responsiveness

We used the standardized response mean (SRM) and effect size (ES) as the indices of responsiveness for the MoCA. The SRM is defined as the change in mean scores divided by the standard deviation (SD) of the change scores between 2 measurement points (i.e., preintervention and postintervention) [13]. The ES is defined as the change in mean scores divided by the SD of the scores at baseline (i.e., preintervention). According to the Cohen criteria, the values of SRM or ES are classified as large (>0.8), moderate (0.5-0.8), and small (<0.5) [20].

#### 2.4.2. Minimal Clinically Important Difference

The distribution-based and anchor-based approaches were used to estimate the MCIDs of the MoCA. On the basis of the Cohen ES benchmark, the distribution-based MCID estimate is suggested to adopt a threshold value of 0.5 SD [21]. According to the anchor-based approach, we compared the change scores of the MoCA with an external anchor, which, in the current study, was the perceived recovery score of the SIS 3.0. The MCID estimates were calculated as the mean change scores of the MoCA, corresponding to patients with a 10% to 15% change on the perceived recovery score, who were defined as having achieved the MCID in a previous study [22].

#### 2.4.3. Validity

The concurrent and predictive validity of the MoCA was calculated using the Spearman rho (*ρ*) test to calculate the correlations with the SIS 3.0. For concurrent validity, the preintervention and postintervention scores of the MoCA were correlated with their respective preintervention and postintervention scores on the criterion measure (i.e., SIS 3.0). For the assessment of predictive validity, the preintervention values of the MoCA were correlated with the postintervention values on the criterion measure. The strength of correlations was defined as excellent (>0.75), good (0.50-0.75), fair (0.25-0.50), and low (≤0.25).

## 3. Results

The study initially included 69 eligible stroke patients, but 4 participants were excluded because of incomplete data, leaving 65 patients for the analysis. Demographic and clinical characteristics of the participants are reported in Table 1. The patients included in the study had significantly higher MoCA scores at postintervention than at preintervention (25.95 ± 3.77 vs. 24.42 ± 4.29, *p* < .001).

### 3.1. Responsiveness and MCID

The results on the responsiveness and MCID estimates of the MoCA are presented in Table 2. The ES approach revealed small responsiveness (0.37), whereas the SRM approach indicated moderate responsiveness (0.67). The anchor-based MCID estimated was 1.22, which was calculated as the mean MoCA change score of 23 participants reaching a change of 10% to 15% on the SIS-perceived recovery score. The distribution-based MCID, according to a 0.5 SD of the baseline, was 2.15. The MCIDs were exceeded by 33 participants (50.77%) based on the anchor-based approach and by 20 participants (30.77%) based on the distribution-based approach.

### 3.2. Criterion Validity

The criterion validity of the MoCA was assessed by the correlations of the MoCA total scores and subscale scores with the criterion measure because previous research has demonstrated that the types of cognitive functions may vary among stroke patients (El Hachioui [23]) and that different cognitive domains may differentially contribute to functions related to the quality of life [24].

#### 3.2.1. Preintervention and Postintervention Concurrent Validity

The preintervention and postintervention Spearman correlation coefficients between the MoCA total and subscale scores and the criterion measure (i.e., SIS 3.0) are provided in Table 3. The total score and the attention, language, and abstraction subscales of the MoCA demonstrated fair to good preintervention and postintervention concurrent validity with the SIS communication subscale (*ρ* = 0.335 − 0.523, *p* < .01), whereas fair concurrent validity with the SIS communication subscale was found for MoCA subscales of naming (*ρ* = 0.476, *p* < .01) and delayed recall (*ρ* = 0.409, *p* < .01) only at postintervention. For the SIS memory subscale, the concurrent validity was low to fair with the MoCA total score, naming, and attention at postintervention (*ρ* = .247 − .284, *p* < .05) and was fair with the MoCA abstraction at preintervention (*ρ* = 0.434, *p* < .01) and postintervention (*ρ* = 0.308, *p* < .05). SIS social participation had fair postintervention concurrent validity with the MoCA total score (*ρ* = 0.275, *p* < .05) and subscales of attention (*ρ* = 0.321, *p* < .01), language (*ρ* = 0.244, *p* < .05), and delayed recall (*ρ* = 0.297, *p* < .05).

#### 3.2.2. Predictive Validity

As summarized in Table 4, the total score and the naming, attention, language, and abstraction subscales of the MoCA at preintervention exhibited fair to good predictive validity with the SIS communication subscale at postintervention (*ρ* = 0.286 − 0.527, *p* < .05). In addition, the MoCA total score, attention, and abstraction were able to predict the SIS memory (total score: *ρ* = 0.296, *p* < .05; attention: *ρ* = 0.321, *p* < .01; and abstraction: *ρ* = 0.445, *p* < .01). The MoCA total score and delayed recall showed fair predictive validity with the social participation subscale (*ρ* = 0.321 − 0.387, *p* < .01). The MoCA abstraction subscale was able to fairly predict the SIS-ADL score (*ρ* = 0.257, *p* < .05).

## 4. Discussion

To the best of our knowledge, this is the first study to investigate the MCID of the MoCA in a stroke population. This study also contributes to improved understanding of the responsiveness and criterion validity of the MoCA in stroke survivors receiving rehabilitative therapy. Our results lent support to the metric soundness of the MoCA in stroke rehabilitation.

The current study assessed the responsiveness of the MoCA in a sample of chronic stroke survivors with more than 6 months (mean, 20.23 months) after stroke onset. Our study showed that the MoCA was moderately responsive to recovery changes in stroke survivors based on the SRM index. Previous research established moderate responsiveness of the MoCA as observed in a subacute stroke population (mean time after stroke onset of 30.8 days) [14]. The current finding indicated that the MoCA is responsive to change at 6 months or above after stroke onset. However, a number of factors need to be noted in interpreting the findings. Examples include the use of different responsiveness indices and rehabilitation interventions, as well as patients' earlier exposure to the MoCA (e.g., experiences of receiving the MoCA assessment before participating in this study) [14]. Future research with a debriefing about the previous experience of receiving cognitive evaluation would be helpful to elucidate the issues.

No previous studies have investigated the MCID of the MoCA in stroke survivors undergoing rehabilitative therapy. In the present study, the MCID values were estimated to be 1.22 and 2.15 according to the anchor-based and distribution-based methods, respectively. We believe that stroke survivors who achieve the threshold value may possibly experience a clinically important change (Wong et al., 2017). In addition to the MCID values, this study showed that 50.77% and 30.77% of the stroke survivors exceeded the anchor-based and distribution-based MCIDs, respectively. Several factors may be associated with the estimation of the MCID values, including age, type of stroke, baseline severity, and intensity of rehabilitative intervention. Future studies are warranted to explore these factors that may be related to the estimation of MCID.

Regarding the concurrent validity, our study showed that the total score and the attention, language, and abstraction subscales of the MoCA had acceptable relationships with the SIS communication subscale at preintervention. After the 4- to 5-week rehabilitative therapy, fair to good correlations were observed between the MoCA (including the total score and subtests of naming, attention, language, abstraction, and delayed recall) and the SIS communication subscale. These findings are supported by previous studies (El Hachioui [23, 25]) that indicated the overlap between cognitive and language domains by investigating the relationships between cognitive deficits (e.g., attention, memory, naming, and abstraction reasoning) and language/communication function in patients with poststroke aphasia. The associations between the MoCA and the SIS communication subscale were also observed to increase in stroke patients at postintervention. It is possible that the intervention improves cognitive functioning (e.g., attention), which indirectly enhances language/communication performance in patients (e.g., [26]) or helps patients perform better on the MoCA.

In addition to the SIS communication, we found that other cognitive functions measured in the MoCA had acceptable relationships with the SIS after the intervention. For instance, the MoCA total score, naming, attention, and abstraction are associated with the SIS memory subscale, and pronounced relationships were observed between the MoCA total score, attention, language, and delayed recall and the SIS social participation subscale. Previous research reported that cognitive deficits are associated with difficulties in ADL performance (e.g., social participation) and decreased quality of life [24]. Our present study indicates that the 4- to 5-week intervention had some positive effects on cognition functioning that is important for recovery from stroke.

We found that the MoCA total score at preintervention had fair predictive validity for the memory, communication, and social participation domains of the SIS at postintervention, indicating that better cognitive functioning among stroke patients before intervention is associated with more favorable rehabilitation outcome in memory, communication, and social participation. Our finding is similar to that in the study of Chen et al. [27], which reported the associations of cognition (measured by the MMSE) with most domains of health-related quality of life (measured by the SIS 3.0) and the predictive power of cognition for memory and communication. However, different from the Chen et al. study [27], the current study observed a significant relationship between cognition at preintervention and social participation at postintervention. The inconsistent results may be due to the use of different assessment tools and treatment protocols in the studies. In addition, the stage after stroke when the assessments are delivered could lead to different degrees of predictive validity. Future research with a longitudinal design would be helpful to address the possibility.

The current study has advanced the research on the psychometric and clinimetric properties of the MoCA. Several limitations warrant consideration. First, the psychometric and clinimetric properties of the MoCA may be influenced by participant characteristics (age, ethnic disparities, etc.). The present findings (e.g., the MCID values estimated in this study) may not be applicable to study samples with different demographic characteristics and cultural background. Second, the study did not analyze data relevant to lesion characteristics in stroke patients. Because the location of the stroke lesion may be associated with various cognitive deficits (El Hachioui [23]), a further study of the lesion effect is warranted when establishing the responsiveness and MCID of the MoCA in stroke patients. Third, because the study only recruited patients with MMSE ≥ 21 (relatively good in baseline MMSE and MoCA performance), future research in survivors with lower cognitive functioning is needed to establish the clinical utility of the MoCA in patients with different cognitive profiles. Fourth, this study excluded those patients unable to follow or understand the instruction of the MoCA. Future research may investigate the possibility of modifying the MoCA instruction (e.g., use of video-assisted modules) to include patients with comprehension deficits for the study.

## 5. Conclusions

The current study examined the responsiveness, MCID, and criterion validity of the MoCA in stroke patients undergoing rehabilitative therapy. Our results showed the acceptable responsiveness and criterion validity of the MoCA in stroke patients. The MCID estimates of the MoCA provide relevant information regarding the evaluation of treatment benefits. The present findings should be interpreted with caution. The factors that may affect the metric properties of the MoCA warrant further scrutiny to validate the findings and extend this present research.

## Figures and Tables

**Table 1:** Demographic and clinical characteristics of the sample.

Variable	Mean ± SD or No. (%) (*N* = 65)
Age (years)	53.54 ± 11.70
Sex	
Male	49 (75.4)
Female	16 (24.6)
Level of education (years)	11.29 ± 4.63
Side of stroke	
Right	40 (61.5)
Left	25 (38.5)
Handedness	
Right	62 (95.4)
Left	3 (4.6)
FMA total score	34.38 ± 9.92
MMSE score	28.12 ± 2.0
MoCA score	
Preintervention	24.42 ± 4.29
Postintervention	25.95 ± 3.77
Months after stroke onset	20.23 ± 13.48
Brunnstrom stage of the UE	
Proximal, median (Q1-Q3)	3 (3-4)
Distal, median (Q1-Q3)	3 (3-4)

Note. FMA: Fugl-Meyer Assessment; MMSE: Mini-Mental State Examination; MoCA: Montreal Cognitive Assessment; SD: standard deviation; UE: upper extremity; Q1-Q3: interquartile range.

**Table 2:** Responsiveness and MCID estimates of the MoCA.

Variable	Value
Responsiveness	
Effect size	0.37
Standardized response mean	0.67
MCID	
Anchor-based: SIS recovery score (10%-15%)	1.22
Distribution-based: 0.5 SD	2.15
Participants who exceeded the MCID, No. (%)	
Anchor-based: SIS recovery score (10%-15%)	33 (50.77)
Distribution-based: 0.5SD	20 (30.77)

Note. MCID: minimal clinically important difference; SIS: Stroke Impact Scale; SD: standard deviation.

**(a):** 

	SIS									
	Strength		Memory		Emotion		Communication		ADL	
	Pre	Post	Pre	Post	Pre	Post	Pre	Post	Pre	Post
MoCA										
Total score	.037	–.024	.114	.247^∗^	–.091	.141	.335^∗∗^	.523^∗∗^	.117	.178
Visuospatial	–.034	–.216	–.008	–.008	–.308^∗^	–.119	.001	–.022	.012	–.174
Naming	.101	–.055	.027	.284^∗^	–.201	.149	.204	.476^∗∗^	.145	.134
Attention	.045	–.023	.184	.255^∗^	.024	.006	.387^∗∗^	.455^∗∗^	.048	.260^∗^
Language	.013	–.087	.093	.233	–.188	–.022	.391^∗∗^	.487^∗∗^	.045	.194
Abstraction	.209	.185	.434^∗∗^	.308^∗^	.031	.113	.454^∗∗^	.397^∗∗^	.221	.171
Delayed recall	–.063	.029	–.009	.143	.023	.247^∗^	.120	.409^∗∗^	.139	.149
Orientation	.116	.095	–.055	–.045	–.071	.075	.098	.024	–.092	.030

**(b):** 

	SIS							
	Mobility		Hand function		Social participation		Physical function	
	Pre	Post	Pre	Post	Pre	Post	Pre	Post
MoCA								
Total score	.084	.130	.187	.034	.132	.275^∗^	.193	.113
Visuospatial	.093	.095	.116	–.133	–.148	–.120	.086	–.169
Naming	.096	.214	.092	–.027	.080	.147	.166	.027
Attention	.049	.096	.080	.096	.069	.321^∗∗^	.107	.139
Language	–.059	.035	.223	.057	.043	.244^∗^	.149	.096
Abstraction	.143	.246^∗^	.189	.058	.084	.120	.258^∗^	.198
Delayed recall	.162	.055	–.009	–.060	.217	.297^∗^	.055	.043
Orientation	–.036	–.062	.285^∗^	.071	–.006	.123	.145	.074

Note. ^∗∗^*p* < 0.01; ^∗^*p* < 0.05; ADL: activity of daily living; Pre: preintervention; Post: postintervention.

**Table 4:** Predictive validity of the MoCA.

	SIS								
	Strength	Memory	Emotion	Communication	ADL	Mobility	Hand function	Social participation	Physical function
MoCA									
Total score	.005	.296^∗^	.107	.507^∗∗^	.193	.111	.152	.321^∗∗^	.189
Visuospatial	–.028	.172	–.066	.118	.028	.077	.007	–.046	.036
Naming	–.028	.142	–.123	.286^∗^	.164	.006	.058	.066	.109
Attention	–.020	.321^∗∗^	.136	.527^∗∗^	.110	.164	–.005	.228	.049
Language	–.092	.197	–.059	.443^∗∗^	.155	.005	.181	.207	.167
Abstraction	.069	.445^∗∗^	.015	.481^∗∗^	.257^∗^	.215	.074	.091	.168
Delayed recall	.064	.185	.177	.242	.147	.092	.066	.387^∗∗^	.110
Orientation	–.037	.012	.099	.158	.008	–.026	.206	.052	.106

Note. ^∗∗^*p* < 0.01; ^∗^*p* < 0.05.

## Data Availability

The data used to support the findings of this study are available from the corresponding author upon request.
